# Prediction of Anticancer Peptides with High Efficacy and Low Toxicity by Hybrid Model Based on 3D Structure of Peptides

**DOI:** 10.3390/ijms22115630

**Published:** 2021-05-26

**Authors:** Yuhong Zhao, Shijing Wang, Wenyi Fei, Yuqi Feng, Le Shen, Xinyu Yang, Min Wang, Min Wu

**Affiliations:** State Key Laboratory of Natural Medicines, School of Life Science and Technology, China Pharmaceutical University, Nanjing 210009, China; zhaoyuhong96@163.com (Y.Z.); wsjwendy@sina.com (S.W.); m15850658512@163.com (W.F.); 3219030694@stu.cpu.edu.cn (Y.F.); sl865070@163.com (L.S.); beiliyashizhu@163.com (X.Y.)

**Keywords:** anticancer peptides, hemolytic peptides, toxic peptides, machine learning, ensemble algorithms, hybrid models, three-dimensional structure, multiple datasets

## Abstract

Recently, anticancer peptides (ACPs) have emerged as unique and promising therapeutic agents for cancer treatment compared with antibody and small molecule drugs. In addition to experimental methods of ACPs discovery, it is also necessary to develop accurate machine learning models for ACP prediction. In this study, features were extracted from the three-dimensional (3D) structure of peptides to develop the model, compared to most of the previous computational models, which are based on sequence information. In order to develop ACPs with more potency, more selectivity and less toxicity, the model for predicting ACPs, hemolytic peptides and toxic peptides were established by peptides 3D structure separately. Multiple datasets were collected according to whether the peptide sequence was chemically modified. After feature extraction and screening, diverse algorithms were used to build the model. Twelve models with excellent performance (Acc > 90%) in the ACPs mixed datasets were used to form a hybrid model to predict the candidate ACPs, and then the optimal model of hemolytic peptides (Acc = 73.68%) and toxic peptides (Acc = 85.5%) was used for safety prediction. Novel ACPs were found by using those models, and five peptides were randomly selected to determine their anticancer activity and toxic side effects in vitro experiments.

## 1. Introduction

According to the latest cancer statistics, cancer incidence and death rates are increasing year by year [1,2]. Traditional cancer treatment methods mainly include surgery, radiation therapy, chemical drugs and macromolecular targeted drugs. However, cancer treatment continues to face the challenge of increasing resistance to chemical and receptor-targeted anticancer drugs. Researchers identified a class of bioactive peptides with antitumor activity, named anticancer peptides (ACPs), which are found in a wide range of organisms, including mammals, amphibians, insects, plants and microorganisms [3]. As a new potential drug for cancer treatment, ACPs are cationic amphiphilic peptides with a length of about 5–50 amino acids, which are characterized by a simple structure, easily synthesized, easily modified chemically and less immunogenic [4]. Owing to the increased proportion of phosphatidylserine (negatively charged) on the surface of cancer cells compared to normal cells, cationic amphiphilic peptides may be an effective and highly selective antitumor drug. The antitumor mechanisms of ACPs can be divided into two types: selective membrane destruction and non-membrane dissolution, which include inhibition of angiogenesis and promotion of tumor cell apoptosis [5]. Despite these advantages, ACPs still face challenges before becoming effective clinical agents, such as poor stability, hemolysis and toxicity to normal tissue cells. The stability of peptides can be improved on various ways, including incorporation of unnatural amino acids, cyclization and modification of the chemical skeleton [6]. Therefore, it is essential to develop methods to identify safer and more effective ACPs.

Machine learning derived from artificial intelligence and statistics is one of the key research directions in the field of data analysis at present. The application of machine learning algorithms in drug research and development greatly speeds up the process of early drug screening. Basic machine learning algorithms includes naive Bayes (NB), support vector machine (SVM), random forests (RF), K nearest neighbor (KNN), artificial neural networks (ANNs), ensemble algorithms, etc. Due to identifying potential novel ACPs using experimental methods require considerable time and expense, in order to aid wet-laboratory researchers discover novel ACPs, various machine learning approaches are used to ACPs recognition [7].

In the past decades, there have been many significant studies on the prediction of ACPs based on sequence features. In the beginning, Tyagi et al. [8] used the SVM method to construct an online predictor AntiCP based on amino acid composition, dipeptide composition and binary profile pattern. Then, Hajisharififi et al. [9] developed a model of SVM based on Chou’s pseudo-amino acid composition (PseAAC) and local alignment kernel. After that, researchers have established many predictors, for example, ACPP [10], iACP [11], MLACP [12], iACP-GAEnsC [13], ACPred-FL [14], SAP [15], TargetACP [16], ACPred [17], mACPpred [18], ACPred-Fuse [19], PTPD [20], ACP-DL [21], PEPred-suite [22], AntiCP 2.0 [23], CancerGram [24], DeepACP [25] and ENNAACT [26], most of them adopt diverse feature extraction methods combined with various machine learning algorithms. All of the above predictors performed well in distinguishing between ACPs and non-ACPs. Additionally, in order to screen safe candidate peptide drugs, some prediction models of toxic peptide and hemolytic peptide have been developed successively [27,28,29,30,31,32,33].

**Figure 1:**
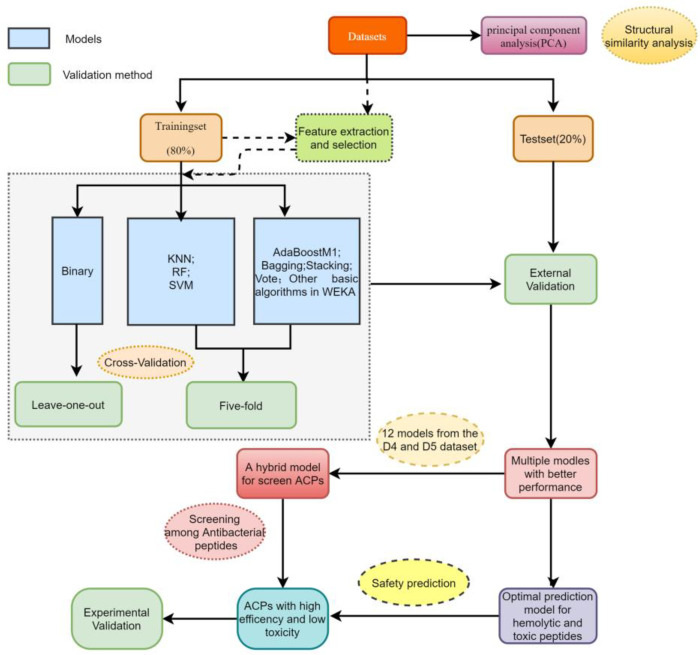
The flowchart describes the overall implementation approach for screening high-efficiency and low-toxicity ACPs.

Although tremendous advances in the field of ACPs prediction, these approaches are almost entirely based on peptide sequences of feature extraction and use very similar datasets. In addition to the basic characteristics of peptide sequence, the 3D structure of ACPs plays a key role in inhibiting tumor cell proliferation. SATPdb [34] is a database annotating the tertiary structure of various therapeutic peptides using PEstrMOD [35], homology modeling [36] and I-TASSER Suite [37] methods, which provides a data basis of structure-based activity and toxicity prediction of ACPs. Consequently, in this study, we collected five datasets of 3D structure of ACPs and used a variety of machine learning algorithms to establish the model. Similarly, the tertiary structure datasets of hemolytic peptides and toxic peptides were collected to construct models for predicting the safety of ACPs. Then, 12 algorithms with the better performance were selected from the two ACPs datasets to form a mixed model to screen ACPs from antimicrobial peptides (AMPs). The optimal prediction models of hemolytic peptides and toxic peptides were used to ensure the safety of candidate ACPs (Figure 1). To our knowledge, this is the first reported method to simultaneously predict ACPs activity, hemolysis and toxicity based on the 3D structure of peptides. Novel ACPs were discovered by the above methods, and their anticancer efficacy and toxicity were evaluated by in vitro experiments.

## 2. Results

### 2.1. The Composition of Multiple Datasets

**Figure 2:**
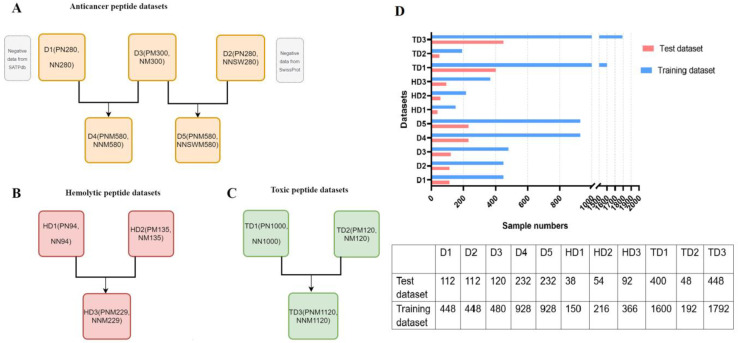
Composition of multiple datasets. (**A**) Number of positive and negative samples in five datasets of anticancer peptides. PN, NN, NNSW, PM and NM indicate positive natural peptides, negative natural peptides, negative natural peptides derived from SwissProt, positive chemically modified peptides and negative chemically modified peptides, respectively. PNM represents a positive dataset of mixed natural peptides and chemically modified peptides. NNM implies a negative dataset of mixed natural peptides and chemically modified peptides. (**B**) The number distribution of positive and negative samples of three hemolytic peptides datasets. (**C**) The number distribution of positive and negative samples in three toxic peptide datasets. (**D**) Data splitting of all datasets. Blue indicates training set; pink indicates test set.

The positive and negative sample compositions of five ACPs datasets, three hemolytic peptides datasets and three toxic peptides datasets are shown in Figure 2A–C. Figure 2D shows the training set and test set division of all datasets. The above datasets are detailed in Materials and Methods 4.1 Datasets.

### 2.2. Structural Similarity Analysis

**Figure 3:**
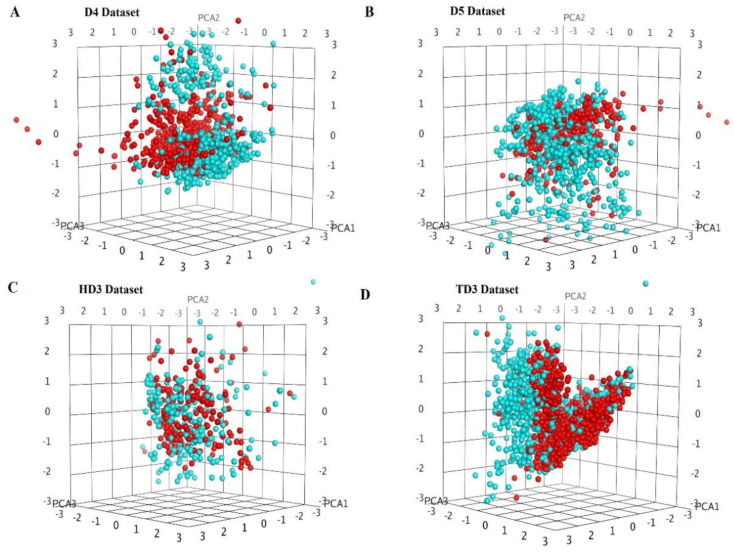
Structural similarity analysis based on principal component analysis. (**A**) ACPs mixed dataset D4. (**B**) ACPs mixed dataset D5 (data of negative natural peptides were obtained from Swissprot). (**C**) Hemolytic peptide mixed dataset HD3. (**D**) Toxic peptide mixed dataset TD3. Blue indicates negative data and red indicates positive data.

The first three principal components were extracted from structural features for similarity analysis. The positive data and negative data of the ACPs datasets were obviously distinguished in the three-dimensional space. The concentrated distribution of polypeptides with anticancer activity indicated that the extracted features were very effective. In particular, the chemically modified peptide dataset D3 performed better than other ACPs datasets. The distinction between positive and negative data was not obvious in the hemolytic peptide datasets, and the data distribution was relatively less concentrated. Toxic peptides have very similar features and can clearly distinguish between positive and negative data. Structural similarity analysis of mixed peptide datasets is shown in Figure 3, and other datasets in Figure S1.

### 2.3. Development of Models on ACPs Dataset

**Figure 4:**
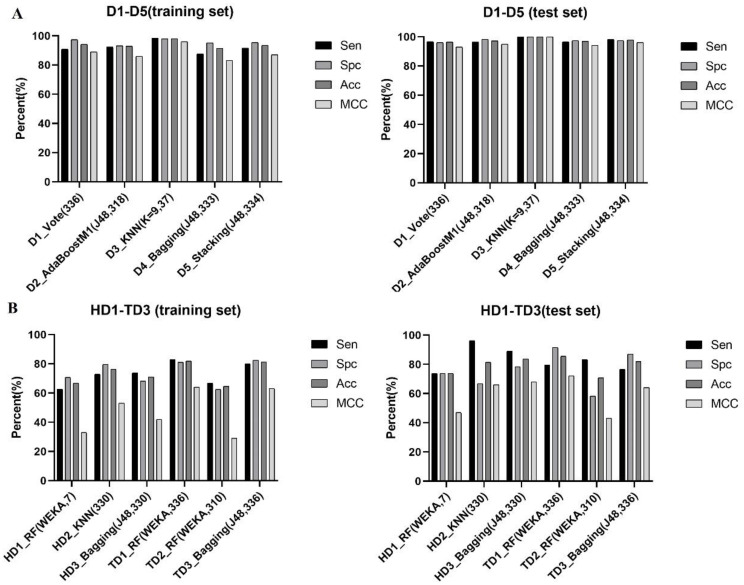
The optimal model for each dataset. (**A**) The performance of the optimal models of 5 ACPs datasets on the training set and the test set. (**B**) Performance of optimal models of hemolytic peptide and toxic peptide datasets on training sets and test sets.

In this study, five ACP datasets were collected to explore the influence of different datasets on model construction. The model of D2 performs better than the model of D1 in the natural peptide dataset (Table 1, Figure 4A), which indicates that ACPs and AMPs have similar structures, and their differentiation is slightly weaker than that of ACPs and peptides derived from Swissprot. The model performance of chemically modified peptide dataset D3 was better than that of natural peptide dataset D1 and D2, illustrating that the feature extraction method in this study was easier to extract the chemically modified features of peptides, so the chemically modified ACPs were easier to distinguish from modified AMPs. Similarly, the model of mixed peptide dataset D5 was superior to the model of D4 (Table 2). Overall, it is crucial to select the appropriate dataset when constructing the prediction model.

#### 2.3.1. D1 Dataset

A total of 306 descriptors were obtained by preliminary feature screening in D1 dataset, and 24 descriptors were obtained by further feature selection method in WEKA (Table S1). A variety of algorithms were selected for model construction based on 306 and 24 features respectively. Then, the optimal model is selected from the classical algorithms Binary, RF, SVM and KNN. From WEKA ensemble algorithm (AdaBoostM1, Bagging, Stacking and Vote) select the optimal model. In the D1 dataset, the best performing model was the Vote algorithm based on 306 features, with accuracy of 94.2% and MCC of 0.89 in the training set and accuracy of 96.43% and MCC of 0.93 in the test set.

#### 2.3.2. D2 Dataset

A total of 318 descriptors were obtained by preliminary screening in D2 dataset, and 20 descriptors were obtained by further feature screening in WEKA (Table S3). In addition, the dataset has 148 descriptors recommended by the MOE descriptor calculation module (Table S2). Same as the D1 dataset, 6 models with better performance were obtained after model construction and selection. The two models with the best performance are AdaBoostM1 algorithm with J48 primary learner, and the selected features are 318 and 20, respectively. It achieved accuracy of 92.84% and MCC of 0.86 on the training set, and accuracy of 97.32% and MCC of 0.95 on the test set.

#### 2.3.3. D3 Dataset

A total of 332 features were selected from the chemically modified peptide dataset D3 after preliminary screening, and the remaining 13 features were further screened by WEKA (Table S5). Furthermore, the dataset has 37 suggested descriptors in the MOE (Table S4). After model construction and selection, five models with excellent performance were obtained. Among them, the model with the best performance is the classical algorithm KNN (k = 9) based on 37 features. It achieved the highest accuracy of 100%, MCC of 1 on the test dataset and accuracy of 98.13%, MCC of 0.96 on the training dataset.

#### 2.3.4. D4 Dataset

A total of 333 descriptors were selected from the mixed peptide dataset D4, and 14 descriptors were obtained by further feature selection by WEKA (Table S7). In addition, the dataset has nine suggested descriptors in the MOE (Table S6). Six models with excellent performance were obtained through model construction and selection. The Bagging algorithm based on 333 features with J48 as the primary classifier has the best performance. It achieved accuracy of 91.49% and MCC of 0.83 on the training set, and accuracy of 96.98% and MCC of 0.94 on the test set (Table 2, Figure 4A).

#### 2.3.5. D5 Dataset

In the mixed dataset D5, 334 features were selected preliminarily and 17 features were further selected in WEKA (Table S9). Moreover, there were 19 suggested descriptors in the MOE (Table S8). Six models with better performance were obtained through model development and screening. The optimal model was based on 334 descriptors developed using J48 as a secondary classifier of Stacking algorithm, with an accuracy of 93.53% and MCC of 0.87 (training set), and accuracy of 96.98% and MCC of 0.94 (test set) (Table 2, Figure 4A).

Selecting low-dimensional features in each dataset performed as well as models constructed with high-dimensional features. These results suggest that high quality models can be developed as long as there are key features. On D1, D2, D4 and D5 data sets, the performance of the ensemble algorithm was relatively slightly better than that of the classical algorithm. In the five datasets of ACPs, all the models screened above showed excellent internal stability and external predictability.

### 2.4. Models Developed on Hemolytic Peptide Dataset

In the natural dataset HD1 of hemolytic peptide, 308 descriptors were selected preliminarily, and the remaining 7 descriptors were further selected by WEKA (Table S10). The optimal model was the RF algorithm derived from WEKA based on 7 descriptors, with an accuracy of 66.67% and MCC of 0.33 (training set) and accuracy of 73.68% and MCC of 0.47 (test set). In the chemically modified peptide dataset HD2, there were 330 features after initial screening, and 18 features were further screened by WEKA (Table S11). The model with the best performance is the KNN algorithm based on 330 features. It achieved accuracy of 76.38% and MCC of 0.53 on the training set, and accuracy of 81.48% and MCC of 0.66 on the test set (Table 3, Figure 4B). In the mixed peptide data set HD3, 330 features were screened for the first time and 23 features were further screened by WEKA (Table S12). The optimal model is the Bagging algorithm based on 330 descriptors with J48 as the primary learner, with an accuracy of 71.04% and MCC of 0.42 (training set), and accuracy of 83.7% and MCC of 0.68 (test set). In the hemolytic peptide dataset, the model chemically modified peptides developed based on the 3D structure features are superior to the natural peptides. The ensemble algorithm derived from WEKA performed better than the classical algorithm. The optimal models in the three hemolytic peptide datasets had good fit in the training set and good generalization ability in the test set.

### 2.5. Models Developed on Toxic Peptide Dataset

In the natural toxic peptide dataset TD1, there were 336 features after preliminary screening and 22 features were further selected by WEKA (Table S13). The best performing model was the RF algorithm derived from WEKA based on 336 features, with accuracy of 81.94% and MCC of 0.64 in the training set and accuracy of 85.5% and MCC of 0.72 in the test set (Table 4, Figure 4B). In the chemically modified peptide dataset TD2, 310 descriptors were selected for the first time, and the remaining 15 descriptors were further screened by WEKA (Table S14). The optimal model was the RF algorithm derived from WEKA based on the 310 features. It achieved an accuracy of 64.58% and MCC of 0.29 on the training set and an accuracy of 70.83% and MCC of 0.43 on the test set. In the mixed peptide dataset TD3, 336 descriptors were obtained by preliminary screening and 27 descriptors were obtained by further selecting by WEKA (Table S15). The optimal model was the Bagging algorithm based on 336 features with J48 as the primary learner, with an accuracy of 81.31% and MCC of 0.63 (training set), and accuracy of 81.92% and MCC of 0.64 (test set). In the toxic peptide dataset, the performance of the model developed by the features extracted from chemically modified peptides was weaker than that of natural peptides, possibly due to similar chemical modification structures on toxic peptides and AMPs. Additionally, the model developed by the ensemble algorithm is superior to the classical algorithm. The three optimal models have good internal fitting and external predictability.

### 2.6. Screening of Candidate ACPs

**Figure 5:**
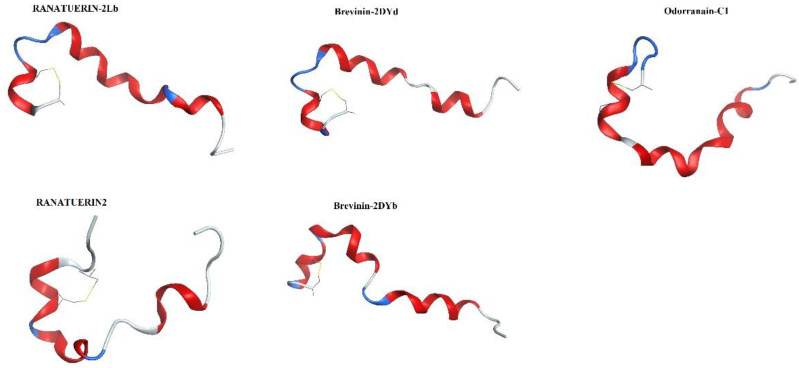
Three-dimensional structure of 5 candidate ACPs.

A total of 1294 AMPs were collected as a dataset to screen peptides with anticancer activity. Since the dataset included chemically modified peptides and natural peptides, we used the models in the ACPs mixed dataset D4 and D5 for ACPs prediction. As shown in Table 2, the 12 models developed in the D4 and D5 data sets were all excellent. In order to increase the accuracy of the prediction model, we tried to combine the prediction results of the 12 models to select ACPs. The screening criterion of the mixed model was that the ACPs were determined only when the predicted results of more than nine (including 9) models were positive. A total of 83 candidate ACPs were screened by the above method. Due to almost all of the 83 anticancer peptides being natural peptides, the optimal model of hemolytic peptide HD1 and toxic peptide TD1 was selected for safety prediction. In the end, we obtained 41 candidate ACPs whose hemolysis and toxicity were predicted to be negative. A total of 5 peptides were randomly selected from 41 candidate anticancer peptides for experimental verification. The sequence and structural information of the five polypeptides are shown in Table 5 and Figure 5, and the information of 41 candidate ACPs is listed in Table S16.

### 2.7. Experimental Verification

**Figure 6:**
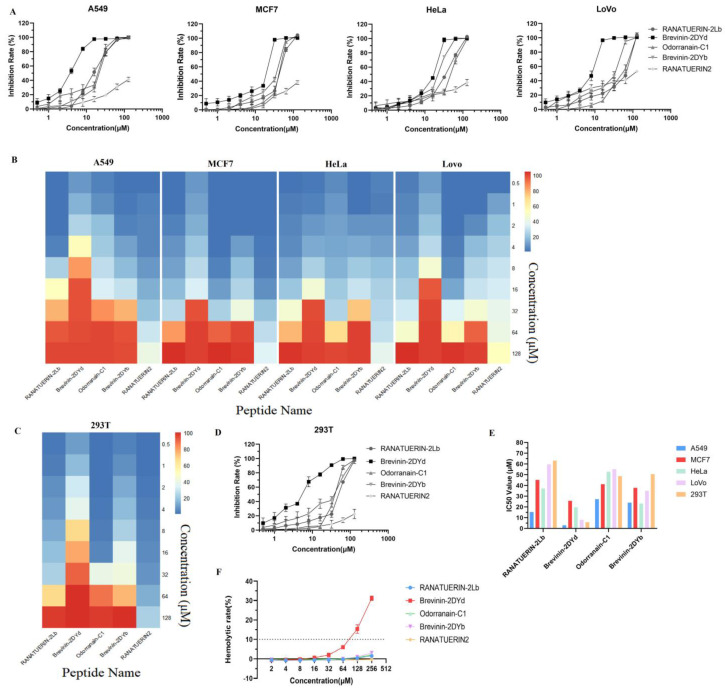
In vitro anticancer activity and toxicity of 5 peptides. (**A**) The inhibition rates of 5 peptides against 4 types of cancer cells (A549, MCF7, HeLa and LoVo) were calculated at different concentrations. (**B**) The heat map plot was used to show the inhibition rate of 5 peptides on diverse cancer cells. Inhibition rate is the average of three replicates. (**C**,**D**) Inhibition rate of human embryonic kidney cells 293T under 5 peptides concentrations. (**E**) The IC50 values of 4 peptides on 5 cell lines. (**F**) Hemolytic activities of selected ACPs at different concentrations.

The inhibition rates of several cancer cell lines in the CancerPPD were analyzed statistically [38], with median activity (EC/IC/LC50 (μM)) ranging from 17 ± 3 μM to 53 ± 9 μM. Therefore, compared with the ACPs in cancerPPD, the candidate five peptides showed anticancer activity when the IC50 value was less than 50 μM. The anticancer action of the five purified peptides was tested on A549, MCF7, HeLa and LoVo cancer lines, and the concentration that inhibits half of the cell growth was calculated (half-inhibitory concentration [IC50]). Four of the five peptides exhibited anticancer activity and inhibited the growth of at least two types of cancer cells (Figure 6A,B,E, Table 6), which demonstrated the efficacy of the hybrid ACPs model to a certain extent. All the four peptides (Ranatuerin-2Lb, Brevinin-2DYd, Odorranain-C1 and Brevinin-2DYb) had effective killing effects on lung cancer cell A549, especially the IC50 of Brevinin-2DYd and Ranatuerin-2Lb were 2.975 μM and 15.32 μM, respectively. Brevinin-2DYd showed significant inhibitory effects on four cancer cells relative to other peptides.

To verify the effectiveness of the toxic peptide model, we selected human embryonic kidney cells 293T to test the inhibitory effect of candidate ACPs on non-cancerous cells (Figure 6C–E, Table 6). Similar to the division of ACPs activity, peptides were considered to have low toxicity when the IC50 value exceeded 50 μM. Three (RANATUERIN-2Lb, Brevinin-2DYb, RANATUERIN2) of the five peptides showed low toxicity to 293T cells, Odorranain-C1 exhibited certain toxicity and Brevinin-2DYd showed the highest toxicity. To a certain extent, the experimental results suggested that the prediction model of toxic peptide had a certain predictability, which was consistent with the model verification results.

We further reported the hemolytic activity of the 5 peptides, the hemolytic rate of 100 μM peptides was more than 10% as the classification standard of hemolysis, and there was no hemolysis in the 4 peptides (RANATUERIN-2Lb, Odorranain-C1, Brevinin-2DYb and RANATUERIN2) and moderate hemolysis in Brevinin-2DYd (Figure 6F, Table 7). To some extent, these results indicate that the developed RF model derived from WEKA is effective in the prediction of hemolytic peptides.

In summary, these results show that RANATUERIN-2Lb and Brevinin-2DYb are potential ACPs considering the anticancer activity and safety of peptides. RANATUERIN-2Lb and Brevinin-2DYb have selective killing effect on lung cancer A549 cells relative to 293T cells, and can be developed as potential candidate drugs for lung cancer. These experiments are only to validate the practicability of the developed ACPs, hemolytic peptides and cytotoxic peptides models, and more systematic experiments are needed to further develop ACP drugs.

## 3. Discussion

The purpose of this study was to construct models for predicting anticancer activity and safety of peptides based on their 3D structures, and to collect different datasets to compare the differences between the models developed by natural peptides and chemically modified peptides. In the ACPs datasets, KNN and RF of the classical algorithm are excellent, while AdaBoostM1, Bagging, Vote and Stacking of the ensemble algorithm show high accuracy. Compared with the previous model prediction methods for ACPs [10,11,12,13,14,15,16,17,18,19,20,21,22,23,24,25,26], features were extracted based on the 3D structure of peptides for the first time, which can be used as a supplementary method for the prediction of ACPs. The extraction of features from the 3D structure of peptides can better reflect the state of peptides in organisms and analyze the properties of peptides from different perspectives. Compared with the existing prediction models such as ENNAACT [26] and AntiCP 2.0 [23], the ACPs model developed by us is very robust only by its accuracy and MCC. However, due consideration has to be given to the differences caused by different methods of extracting features, so it is uncertain whether our model is superior to other models. The results of the test set of the model constructed in the ACPs dataset were slightly better than those of the training set, which may be due to the fact that the sample size of the test set is small, and the characteristics of positive and negative samples are clearly distinguished. Meanwhile, the ensemble algorithm adopted can prevent the overfitting of the training set, so as to maintain the good fit of the model and improve the external generalization ability as much as possible. Some peptide prediction models also show similar results, such as the model built by Piyush. et al., using the SVM algorithm [23], and the model developed by Vishuda. et al., using the RF algorithm [39].

Similarly, it is the first time to predict both hemolysis and toxicity of anticancer peptides while predicting their activities. Referred to the model developed by Vinod et al. using a similar approach [32], our model constructed in the hemolytic peptide chemical modification dataset HD2 achieved good results. However, our model in the toxic peptide dataset and the existing model uses different methods of feature extraction, so it is hard to compare the model performance. Multiple datasets collected in this study, the ACPs and hemolytic peptides collected were verified by experiments, while the data of toxic peptide datasets are all kinds of peptides with high toxicity, not just toxicity to non-cancerous cells. Toxic peptides act on non-cancer cells through a variety of mechanisms including interactions with specific ion channels, enzymes, mitochondria and membrane components. Therefore, the model for predicting hemolysis and toxicity developed in this study can be used to predict the toxic and side effects of various peptides. In order to further improve the accuracy of toxicity prediction, it is necessary to collect relatively appropriate datasets in the future.

In the classical peptide databases APD3 [40], CAMP_R3_ [41], DRAMP 2.0 [42] and SATPdb [34], the number of ACPs accounts for about 10% of AMPs. Thus, some AMPs exhibited anticancer activity [43], a candidate dataset containing 1294 AMPs was collected to screen novel ACPs. The hybrid ACPs model and the optimal hemolytic and toxicity prediction model developed in this study were applied to the candidate dataset to screen out 41 candidate ACPs and 5 of them were randomly selected for experimental verification. To a certain extent, the results of this study indicate that the mixed model for screening ACPs had excellent practicability, the hemolytic peptide model also had good applicability, and the toxic peptide model had a certain predictability. Taken together, these results suggest that RANATUERIN-2Lb and Brevinin-2DYb have both anticancer activity and safety, and are expected to be developed as candidate ACPs drugs. The method proposed in this study provides a new idea for predicting the activity and safety of ACPs. Although we obtained good results by selecting peptides with good predicted effects for experimental verification, and had a certain effect on predicting candidate ACPs drugs, the experimental verification method was not perfect enough from the perspective of model verification. As they have suggested, the peptides with the worst ensemble prediction results should be selected for further experiments to fully demonstrate the efficacy of the developed model.

Overall, we developed models for predicting the activity and safety of ACPs based on the 3D structure of peptides, and identified two novel candidate ACPs. Although the present method is restricted by the time consuming of the structure prediction step, it is still a powerful complement to the method of building models based on sequence features. With the development of computational biology, more and more 3D structure-based peptide activity prediction methods are expected to be developed and used widely. In order to verify whether the model is reliable, we randomly selected 5 peptides for experiment, and we will continue to conduct experiments on the remaining 36 candidate peptides to select the ACPs with high efficiency and low toxicity in the future. Considering that the structure of the peptide may change when it interacts with the cell, it is important to verify its three-dimensional structure through experiments. At present, the methods of protein crystal structure analysis mainly include nuclear magnetic resonance (NMR), X-ray diffraction imaging (XDI) and cryo-electron microscopy (Cryo-EM) [44]. Due to the limitation of experimental conditions, the 3D structure verification experiment of peptide cannot be carried out, so we hope to improve it in the subsequent studies. In this study, the selection of candidate ACPs mainly focused on natural ACPs, while the general chemically modified ACPs had better effects. Based on the excellent prediction model of chemically modified peptides developed in this paper, chemically modified peptides with high anticancer activity will be screened in future studies.

## 4. Materials and Methods

### 4.1. Datasets

#### 4.1.1. ACPs Datasets

In this study, we created 5 datasets in order to compare the differences between diverse datasets in constructing the prediction model of ACPs. We collected sequence and structure data on experimentally validated ACPs in CancerPPD [45] and SATPdb. Afterwards, natural peptides and chemically modified peptides are classified according to whether there are chemical modifications. Generally, acetylation, amidation, methylation, glycosylation and non-natural residues are counted as chemical modifications [46]. We extracted 435 natural ACPs from CancerPPD and SATPdb, and collected 300 chemically modified ACPs from CancerPPD. In order to remove sequence redundancy in the dataset, CD-Hit [47] was used to delete sequences with more than 85% similarity, and 280 natural ACPs were obtained. The non-anticancer AMPs were selected from SATPdb as non-ACPs, 360 chemically modified non-ACPs were extracted and 471 natural non-ACPs were screened using CD-Hit with sequence identity cut-off of 85%. In addition, we retrieved 356 random peptides from SwissProt [48] Proteins using the following keywords, “not anticancer activity”, “amino acid length range of 5 to 70” and “with 3D structure”, as another natural non-ACPs dataset.

In order to create several balanced datasets, 280 natural ACPs and 280 natural AMPs from the SATPdb were selected as the D1 dataset. The D2 dataset was composed of 280 natural ACPs and 280 random peptides from SwissProt. The D3 dataset was made of 300 chemically modified ACPs and 300 chemically modified AMPs from SATPdb. Subsequently, natural peptides and chemically modified peptides were placed in one dataset to form a mixed dataset, that is, D1 and D3 constitute the mixed dataset D4, and D2 and D3 form the mixed dataset D5 (Figure 2A). The sequence and 3D structures data of the five ACP datasets were shown in Supplementary Materials 2.

#### 4.1.2. Hemolytic Peptides Datasets

The sequence and structure data of hemolytic peptides verified by experiments were obtained from Hemolytik [49] and SATPdb, and natural peptides and chemically modified peptides were collected respectively. Peptides that satisfy one of the following criteria are considered hemolytic peptides, (i) minimum hemolytic concentration (MHC) ≤ 250 μg/mL; (ii) half maximum effective concentration (EC50) or hazardous concentration (HC50) ≤ 100 μM and (iii) >10% hemolytic activity up to 100 μM [28]. Peptides that do not meet the above criteria are selected as non-hemolytic peptides with extremely low hemolysis at relatively high concentrations. Finally, the natural dataset HD1 of 94 hemolytic peptides and 94 non-hemolytic peptides was obtained through CD-Hit screening. Chemically modified peptide dataset HD2 was composed of 135 hemolytic peptides and 135 non-hemolytic peptides, and HD1 and HD2 constituted the mixed dataset HD3 (Figure 2B). Peptide sequences and 3D structures of the three hemolytic peptide datasets were shown in Supplementary Materials 2.

#### 4.1.3. Toxic Peptides Datasets

Similarly, the structure and sequence data of toxic peptides were obtained from the SATPdb, which were divided into natural peptides and chemically modified peptides. The majority of toxic peptides in the SATPdb are peptide toxins from ATDB [50], Tox-Prot [51], ConoServer [52] and DBETH [53], which are usually highly toxic and have a killing effect on many types of cells, including non-cancer cells. Non-toxic AMPs were retrieved from SATPdb as non-toxic peptides, that is, negative datasets. We extracted the natural dataset TD1 of 1000 toxic peptides and 1000 non-toxic peptides by CD-Hit screening. The chemical modification dataset TD2 was formed from 120 toxic peptides and 120 non-toxic peptides, and TD1 and TD2 are mixed into the TD3 dataset (Figure 2C). Peptide sequences and 3D structures of the three toxic peptide datasets were shown in Supplementary Materials 2.

#### 4.1.4. Candidate Datasets

Since some AMPs also have anticancer properties, we collected a candidate dataset that did not include experimentally validated anticancer peptides in the hope of finding novel ACPs. A total of 2024 non-anticancer and non-toxic antibacterial peptides were obtained from the desired functions module of SATPdb. Then, CD-Hit was used to exclude peptides with sequences that were more than 90% similar to those in all study datasets. Finally, 1294 antibacterial peptides with predicted 3D structures were obtained to compose the candidate dataset.

### 4.2. Internal and External Validations

The length of amino acid sequences in all of the above datasets ranged from 5 to 70. Each dataset was randomly divided into two datasets, that is, 80% of the data constituted the training set and the remaining 20% of the data constituted the test set, and the data ratio between positive samples and negative samples in each subset was about 1:1 (Figure 2D). The training set was used to train the model, and the 5-fold cross validation technique was used for internal validation. For external validation, we used a test set to evaluate the performance of the trained model.

### 4.3. Feature Extraction and Selection

Based on the 3D structure of peptides, the global physicochemical descriptor of the 2018 MOE software [54] was used to extract the features related to the structural properties. The peptides of the same category usually have similar features and a prediction model can be built to distinguish them from other categories of peptides by using machine learning algorithms to recognize their common features. The 3D structure of peptides is closer to the real state of its action in living organisms, so the extracted features can better reflect the properties of peptide drugs. MOE descriptors contain 206 2D descriptors, 148 3D descriptors and 88 protein descriptors, resulting in a total of 435 features. These features include volume and molecular shape, surface area, energy-related descriptors, conformation dependent charge descriptors, etc. To avoid data redundancy, descriptors with poor correlation with activity or toxicity should be removed for preliminary features screening. For every dataset, contingency coefficient C, Cramer’s V, entropic uncertainty U and linear correlation R^2^ of each descriptor were calculated in MOE, and descriptors whose numerical value of C, V, U and R^2^ were 0 were deleted. The remaining descriptors are used for model construction. Then, in each dataset we used the WEKA package [55] for feature selection based on the above descriptors. We choose “CfsSubsetEval” as the evaluator and “Best First” as the default parameter of the search method, that is, the forward direction with amount of backtracking, *N* = 5 and the lookup size D = 1 [46]. In addition, after the calculation of C, U, V and R^2^ mentioned above, MOE has recommended descriptors in some datasets and we built the model according to these descriptors.

### 4.4. Structural Similarity Analysis

A principal component analysis (PCA) was performed on the remaining descriptors of each dataset after preliminary feature screening. Then, the first three principal components were selected to perform the structural feature similarity analysis of 3D visualization for each dataset. The more concentrated the data distribution, the higher the similarity of peptide features.

### 4.5. Machine Learning Techniques

In this paper, we adopted a variety of machine learning algorithms to establish the model, which was mainly divided into two categories: classical algorithm and ensemble algorithm (Figure 1). Classical algorithms include Binary, KNN, RF and SVM. The Binary method is an algorithm based on Bayesian statistics, which can build classification models in the QSAR module of MOE software (2018), and uses the LOO (Leave one out) method for internal verification. The KNN algorithm was run in the Windows command window, the value range of k is 1–9. We used Euclidian distance to describe the similarity between samples, and the adjacent samples were classified into one category. Genetic algorithms with population size of 200 and termination algebra of 300 were used to screen models with correct classification rate (CCR) over 0.6. RF and SVM were implemented by using the random Forest and e1071 package in R respectively. RF uses a set of unpruned decision trees and randomly selects a subset of predictors as candidates for splitting tree nodes [56]. The SVM algorithm uses the radial basis kernel (RBK) function to construct the model, and sets two key parameters that cost as 1000 and gamma as 1 × 10^−6^.

Ensemble algorithms mainly include AdaBoostM1 [57], Bagging [58], Stacking [59] and Vote [60] derived from WEKA software (version 3.8.4). AdaBoostM1 chose DecsionStump (DS) as the primary classifier, and DS, J48, SMO and NaiveBayes (NB) were selected respectively to build four algorithms. Bagging selected REPtree as the basic classifier, and REPtree, J48, SMO, NB, RF and IBK were selected to build six models. Stacking selects ZeroR, PART, OneR, J48, RF, IBK, NB and SMO as the basic learners and J48 as the secondary classifier to build an efficient model. Vote combines multiple algorithms and classifies samples according to the average probability of output. In order to obtain the model with excellent performance, the “CVParameterSelection” method was selected in WEKA to optimize the corresponding parameters of the algorithm.

### 4.6. Performance Evaluation

The performance of the evaluation model was represented by the following parameters: sensitivity (Sen), specificity (Spc), accuracy (Acc) and the Matthew correlation coefficient (MCC). The formula is shown below:

(1) $$\mathrm{Sen}=\frac{\mathrm{TP}}{\mathrm{TP}+\mathrm{FN}}\times 100\%$$

(2) $$\mathrm{Spc}=\frac{\mathrm{TN}}{\mathrm{TN}+\mathrm{FP}}\times 100\%$$

(3) $$\mathrm{Acc}=\frac{\mathrm{TP}+\mathrm{TN}}{\mathrm{TP}+\mathrm{FN}+\mathrm{TN}+\mathrm{FP}}\times 100\%$$

(4) $$\mathrm{MCC}=\frac{\mathrm{TP}\times \mathrm{TN}-\mathrm{FP}\times \mathrm{FN}}{\sqrt{\left(\mathrm{TP}+\mathrm{FP})(\mathrm{TP}+\mathrm{FN})(\mathrm{TN}+\mathrm{FP})(\mathrm{TN}+\mathrm{FN}\right)}}$$

where TP, FP, TN and FN stand for the number of true positives, false positives, true negatives and false negatives, respectively.

### 4.7. Materials

Five peptides were randomly selected from the candidate ACPs and synthesized by Changzhou Kanglong Biotech Ltd (Changzhou, China). using 9-fuorenylmethoxy carbonyl (FMOC) solid phase synthesis technology. The synthetic peptide was identified by mass spectrometry (MS), and the purity >95% by high performance liquid chromatography (HPLC). Human breast cancer cell line (MCF7), lung cancer cell line (A549), cervical cancer cell line (HeLa), colorectal adenocarcinoma cell line (LoVo) and human embryonic kidney cells (293T) were preserved in our laboratory. At 37 °C under 5/95% CO_2_/air condition, all cell lines were cultured in Dulbecco’s modified Eagle medium (DMEM) supplemented with 10% (*v/v*) fetal bovine serum (FBS, Gibco, Carlsbad, CA, USA) and 1% (*v/v*) penicillin/streptomycin (Sangon Biotech, Shanghai, China).

### 4.8. Cell Killing Ability Assay In Vitro

Human tumor cell lines MCF7, A549, HeLa and LoVo were used as experimental materials to detect the anticancer activity of peptides by MTT assay [61,62]. In addition, human embryonic kidney cells 293T were also included in the experiment to examine the safety of the peptide against non-cancerous cells. First, the cells were cultured, then the cells were inoculated in 96-well plates (6000 cells per well) and incubated for 24 h, followed by the addition of five peptides (0.5, 1, 2, 4, 8, 16, 32, 64 and 128 μM) at increasing concentrations for 24 h, each concentration was prepared in triplicate. Untreated cells as negative control group incubated with corresponding medium for 24 h. Subsequently, 10 uL, 5 mg/mL MTT (Sangon Biotech, Shanghai, China) solution was added to each well and incubated for 4 h at 37 °C. After the medium was discarded, 150 μL DMSO was added to each well for 10 min to dissolve formazan crystals. Absorbance was measured at 570 and 630 nm using a multiwall plate reader and the inhibition rate was expressed as the mean ± standard deviation (SD) of the triplicate data.

### 4.9. Hemolysis Assay

The 2% sheep red blood cells (SRBC) were purchased from Nanjing Senbeijia Biological Technology Co., Ltd (Nangjing, China). A total of 4 mL of 2% SRBC was taken, rinsed twice with 4 mL of PBS by centrifugation for 5 min at 3000 rpm and the precipitates were resuspended in 4 mL of PBS. We added 100 μL of 2% SRBC to each well of the 96-well plate and then added 100 μL peptide solutions of different concentrations (2, 4, 8, 16, 32, 64, 128 and 256 μM) in triplicate at 37 °C for 1 h. 100 μL of PBS buffer and 100 uL of Triton X-100 0.1% (*w/v*) were mixed with SRBC as negative and positive controls [63], respectively. All samples were centrifuged at 3000 rpm for 5 min, 100 μL supernatant was collected and transferred to a new 96-well plate, and its absorbance was measured at 540 nm. The calculation formula of hemolysis rate is as follows.

(5) $$\%\;\mathrm{Hemolysis}=\frac{{\mathrm{absorbance}}_{\mathrm{sample}}{-\mathrm{absorbance}}_{\mathrm{negative}}}{{\mathrm{absorbance}}_{\mathrm{positive}}{-\mathrm{absorbance}}_{\mathrm{negative}}}\times 100$$

## Figures and Tables

**Table 1:** In the ACPs datasets D1, D2 and D3, excellent models were constructed based on multiple algorithms and different features.

Dataset	Method	Number of Features	Training Dataset				Test Dataset			
			Sen (%)	Spc (%)	Acc (%)	MCC	Sen (%)	Spc (%)	Acc (%)	MCC
D1	Vote	306	90.87	97.38	94.20	0.89	96.72	96.08	96.43	0.93
D1	KNN (k = 9)	24	81.55	91.69	86.59	0.74	72.13	92.16	81.25	0.65
D1	Bagging (J48)	24	83.56	87.77	85.71	0.71	95.08	94.12	94.64	0.89
D2	RF	318	78.10	97.33	89.48	0.79	96.43	98.21	97.32	0.95
D2	AdaBoostM1 (J48)	318	92.41	93.27	92.84	0.86	96.43	98.21	97.32	0.95
D2	RF	148	83.02	94.22	88.80	0.77	96.43	94.64	95.54	0.91
D2	Bagging (SMO)	148	91.52	90.13	90.83	0.82	96.43	96.43	96.43	0.93
D2	KNN	20	92.37	90.58	91.50	0.83	96.43	96.43	96.43	0.93
D2	AdaBoostM1 (J48)	20	92.41	93.27	92.84	0.86	96.43	98.21	97.32	0.95
D3	Bagging (IBK)	332	97.50	97.92	97.71	0.95	100.00	98.33	99.17	0.98
D3	KNN (k = 9)	37	98.38	97.95	98.13	0.96	100.00	100.00	100.00	1
D3	Bagging (SMO)	37	96.25	98.75	97.50	0.95	100.00	98.33	99.17	0.98
D3	KNN (k = 9)	13	96.04	98.79	97.50	0.95	100.00	100.00	100.00	1
D3	AdaBoostM1 (SMO)	13	96.69	98.31	97.49	0.95	100.00	98.33	99.17	0.98

**Table 2:** In the mixed datasets D4 and D5 of ACPs, excellent models were developed based on a variety of algorithms and different features.

Dataset	Method	Number of Features	Training Dataset				Test Dataset			
			Sen (%)	Spc (%)	Acc (%)	MCC	Sen (%)	Spc (%)	Acc (%)	MCC
D4	KNN	333	86.36	94.10	90.30	0.81	96.55	92.24	94.40	0.89
D4	Bagging (J48)	333	87.72	95.26	91.49	0.83	96.55	97.41	96.98	0.94
D4	Binary	9	70.26	92.67	81.47	0.65	96.55	93.10	94.83	0.90
D4	Stacking (J48)	9	85.13	94.18	89.66	0.80	98.28	95.69	96.98	0.94
D4	KNN (k = 9)	14	87.26	94.77	91.05	0.82	96.55	88.79	92.67	0.86
D4	Bagging (J48)	14	87.50	93.97	90.73	0.82	97.41	96.55	96.98	0.94
D5	KNN	334	92.00	93.98	93.00	0.86	98.28	96.55	97.41	0.95
D5	Stacking (J48)	334	91.59	95.47	93.53	0.87	98.28	97.41	97.84	0.96
D5	KNN (k = 9)	19	89.60	93.03	91.28	0.83	99.14	94.83	96.98	0.94
D5	Bagging (REPtree)	19	87.50	92.46	89.98	0.80	98.28	98.28	98.28	0.97
D5	KNN (k = 9)	17	88.40	95.08	91.70	0.84	97.41	92.24	94.83	0.90
D5	Vote	17	87.50	95.47	91.49	0.83	97.41	96.55	96.98	0.94

**Table 3:** In the hemolytic peptide dataset HD1, HD2 and HD3, the better performance model is developed according to various algorithms and different features.

Dataset	Method	Number of Features	Training Dataset				Test Dataset			
			Sen (%)	Spc (%)	Acc (%)	MCC	Sen (%)	Spc (%)	Acc (%)	MCC
HD1	KNN (k = 9)	308	67.12	61.52	64.00	0.29	73.68	57.89	65.79	0.32
HD1	Vote	308	81.33	80.00	80.67	0.61	73.68	57.89	65.79	0.32
HD1	Binary	7	78.67	76.00	77.33	0.55	63.16	57.89	60.53	0.21
HD1	RF (WEKA)	7	62.67	70.67	66.67	0.33	73.68	73.68	73.68	0.47
HD2	KNN	330	72.97	79.60	76.38	0.53	96.29	66.67	81.48	0.66
HD2	Vote	330	95.37	93.52	94.44	0.89	77.78	81.48	79.63	0.59
HD2	RF	18	76.11	65.85	70.35	0.42	88.89	62.96	75.93	0.54
HD2	Vote	18	78.70	60.19	69.44	0.40	77.78	81.48	79.63	0.59
HD3	Bagging (J48)	330	73.77	68.31	71.04	0.42	89.13	78.26	83.70	0.68
HD3	Binary	23	73.22	79.23	76.23	0.53	58.70	73.91	66.30	0.33
HD3	Bagging (RF)	23	72.68	72.13	72.40	0.45	89.13	71.74	80.43	0.62

**Table 4:** In the toxic peptide dataset TD1, TD2 and TD3, the better performance model was established according to different algorithms and different features.

Dataset	Method	Number of Features	Training Dataset				Test Dataset			
			Sen (%)	Spc (%)	Acc (%)	MCC	Sen (%)	Spc (%)	Acc (%)	MCC
TD1	KNN	336	81.89	80.50	81.19	0.62	80.00	77.50	78.75	0.58
TD1	RF (WEKA)	336	82.88	81.00	81.94	0.64	79.50	91.50	85.50	0.72
TD1	KNN (k = 9)	22	84.18	77.57	80.88	0.62	75.00	85.00	80.00	0.60
TD1	Bagging (J48)	22	82.38	82.75	82.56	0.65	79.00	89.50	84.25	0.69
TD2	KNN (k = 9)	310	74.02	59.30	66.63	0.34	45.83	83.33	64.58	0.31
TD2	RF (WEKA)	310	66.67	62.50	64.58	0.29	83.33	58.33	70.83	0.43
TD2	KNN (k = 9)	15	71.13	60.13	65.11	0.32	45.83	75.00	60.42	0.22
TD3	KNN (k = 9)	336	80.00	79.51	79.74	0.60	71.30	75.11	73.21	0.46
TD3	Bagging (J48)	336	80.04	82.57	81.31	0.63	76.68	87.11	81.92	0.64
TD3	Stacking (J48)	27	76.37	82.23	79.30	0.59	74.89	87.56	81.25	0.63

**Table 5:** Sequence information of 5 peptides.

SATPdb ID	Name	Sequence
16563	RANATUERIN-2Lb	GILSSIKGVAKGVAKNVAAQLLDTLKCKITGC
19566	Brevinin-2DYd	GIFDVVKGVLKGVGKNVAGSLLEQLKCKLSGGC
22121	Odorranain-C1	GVLGAVKDLLIGAGKSAAQSVLKTLSCKLSNDC
22355	RANATUERIN2	GLFLDTLKGAAKDVAGKLEGLKCKITGCKLP
27843	Brevinin-2DYb	GLFDVVKGVLKGAGKNVAGSLLEQLKCKLSGGC

**Table 6:** The IC50 values of 5 peptides inhibited the proliferation of 5 kinds of cells in vitro.

IC50 (μM)	RANATUERIN-2Lb (16563)	Brevinin-2DYd (19566)	Odorranain-C1 (22121)	Brevinin-2DYb (27843)	RANATUERIN2 (22355)
A549	15.32	2.975	27.31	24.01	>128
MCF7	45.25	25.74	41.21	37.84	>128
HeLa	37.23	19.69	52.83	23.26	>128
LoVo	59.78	8.05	55.22	35.05	128
293T	63.16	5.832	48.7	50.66	>128

**Table 7:** Hemolytic effect of 5 peptides on 2% sheep erythrocytes.

Peptides	Peptide Concentration (μM)									
	2	4	8	16	32	64	128	256	Negative Control (PBS)	Positive Control (Triton X-100 (0.1%))
RANATUERIN-2Lb (16563)	−	−	−	−	−	−	−	−	−	+
Brevinin-2DYd (19566)	−	−	−	−	−	−	+	+	−	+
Odorranain-C1 (22121)	−	−	−	−	−	−	−	−	−	+
Brevinin-2DYb (27843)	−	−	−	−	−	−	−	−	−	+
RANATUERIN2 (22355)	−	−	−	−	−	−	−	−	−	+

The “+” symbol indicates that the hemolysis rate exceeds 10% when peptides concentration reaches 100 μM. The “−” symbol indicates the hemolysis rate of less than 10% at peptides concentration of 100 μM.

## Data Availability

The data discussed in this publication can be obtained in Supplementary Materials 2 (https://www.mdpi.com/article/10.3390/ijms22115630/s).

## Supplementary Materials

The following are available online at https://www.mdpi.com/article/10.3390/ijms22115630/s.
