# Supplementary material for: Prediction of Anticancer Peptides with High Efficacy and Low Toxicity by Hybrid Model Based on 3D Structure of Peptides
Source: Int J Mol Sci. 2021 May 26;22(11):5630. doi: 10.3390/ijms22115630 (PMC8198792; doi:10.3390/ijms22115630)
Supplement: Supplementary file 1 [file ijms-22-05630-s001.zip › Final Revised Supplement1 untracked (Supplement figures and tables).pdf]

Prediction of anticancer peptides with high efficacy and low toxicity by hybrid model based on 3D structure of peptides

Yuhong Zhao [1], Shijing Wang [1], Wenyi Fei [1], Yuqi Feng [1], Le Shen [1], Xinyu Yang [1], Min Wang [1,*] and Min Wu [1,*]

[1] State Key Laboratory of Natural Medicines, School of Life Science and Technology, China Pharmaceutical University, Nanjing 210009, China; zhaoyuhong96@163.com (Y.Z.); wsjwendy@sina.com (S.W.); m15850658512@163.com (W.F.); 3219030694@stu.cpu.edu.cn (Y.F.); sl865070@163.com (L.S.); beiliyashizhu@163.com (X.Y.)

* Correspondence: minwang@cpu.edu.cn (M. Wang); mickeywu2001@163.com (M. Wu)

Total number of Supplementary Figures: 1
Total number of Supplementary Tables: 16

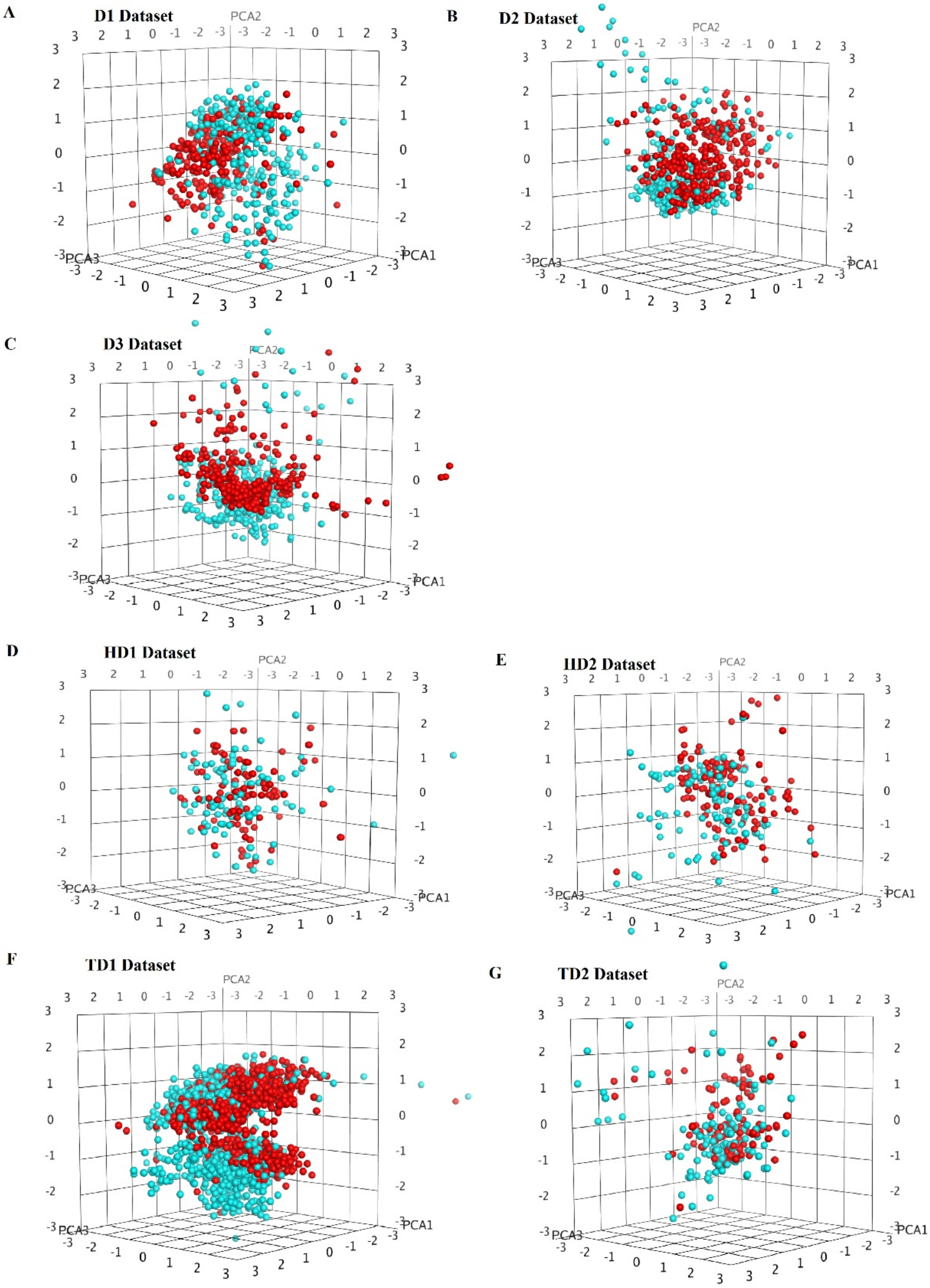

Figure S1. Structural similarity analysis based on principal component analysis. (A) Natural peptide dataset D1 in ACPs. (B) Natural peptide dataset D2 (Negative data were obtained from Swissprot) in ACPs. (C) Chemically modified peptide dataset D3 in ACPs. (D) Natural peptide dataset HD1 in hemolytic peptides. (E) Chemically modified peptide dataset HD2 in hemolytic peptides. (F) Natural peptide dataset TD1 in toxic peptides. (G) Chemically modified peptide dataset TD2 in toxic peptides. Blue indicates negative data, red indicates positive data.

Table S1. 24 descriptors were obtained from D1 dataset through feature screening method of WEKA software.

Number	Feature	Class	Feature Description
1	E	3D	Potential Energy
2	E_ang	3D	Angle Bend Energy
3	E_nb	3D	Non-bonded energy
4	E_str	3D	Bond stretch energy
5	FCharge	2D	Sum of formal charges
6	GCUT_PEOE_0	2D	PEOE Charge GCUT (0/3)
7	GCUT_PEOE_2	2D	PEOE Charge GCUT (2/3)
8	h_pavgQ	2D	Average total charge (pH=7)
9	h_pstates	2D	Entropic state count (pH=7)
10	PEOE_PC+	2D	Total positive partial charge
11	PEOE_VSA+4	2D	Total positive 4 vdw surface area
12	PEOE_VSA-1	2D	Total negative 1 vdw surface area
13	PEOE_VSA-3	2D	Total negative 3 vdw surface area
14	PEOE_VSA-6	2D	Total negative 6 vdw surface area
15	PEOE_VSA_FNEG	2D	Fractional negative vdw surface area
16	PEOE_VSA_FPNEG	2D	Fractional polar negative vdw surface area
17	PEOE_VSA_HYD	2D	Total hydrophobic vdw surface area
18	pmiX	3D	Principal moment of inertia (X)
19	pro_zquadrupole	Protein	Zeta Quadrupole Moment
20	SlogP_VSA4	2D	Bin 4 SlogP (0.10, 0.15]
21	vsurf_CP	3D	Critical packing parameter
22	vsurf_CW1	3D	Capacity factor at -0.2
23	vsurf_Wp3	3D	Polar volume at -1.0
24	vsurf_Wp4	3D	Polar volume at -2.0

Table S2. The D2 dataset has 148 suggested descriptors in the MOE.

Number	Feature	Class	Feature Description
1	apol	2D	Sum of atomic polarizabilities
2	ASA	3D	Water accessible surface area
3	ASA_H	3D	Total hydrophobic surface area
4	a_accc	2D	Number of H-bond acceptor atoms
5	a_count	2D	Number of atoms
6	a_don	2D	Number of H-bond donor atoms
7	a_donacc	2D	Number of H-bond donor + acceptor atoms
8	a_heavy	2D	Number of heavy atoms
9	a_hyd	2D	Number of hydrophobic atoms
10	a_IC	2D	Atom information content (total)
11	a_nC	2D	Number of carbon atoms
12	a_nH	2D	Number of hydrogen atoms
13	a_nN	2D	Number of nitrogen atoms

14	a_nO	2D	Number of oxygen atoms
15	a_nS	2D	Number of sulfur atoms
16	bpol	2D	Difference of bonded atom polarizabilities
17	b_1rotR	2D	Fraction of rotatable single bonds
18	b_count	2D	Number of bonds
19	b_double	2D	Number of double bonds
20	b_heavy	2D	Number of heavy-heavy bonds
21	b_rotR	2D	Fraction of rotatable bonds
22	b_single	2D	Number of single bonds
23	chi0	2D	Atomic connectivity index (order 0)
24	chi0v	2D	Atomic valence connectivity index (order 0)
25	chi0v_C	2D	Carbon valence connectivity index (order 0)
26	chi0_C	2D	Carbon connectivity index (order 0)
27	chi1	2D	Atomic connectivity index (order 1)
28	chi1v	2D	Atomic valence connectivity index (order 1)
29	chi1v_C	2D	Carbon valence connectivity index (order 1)
30	chi1_C	2D	Carbon connectivity index (order 1)
31	chiral	2D	Number of chiral centers
32	chiral_u	2D	Number of unconstrained chiral centers
33	E_sol	3D	Solvation energy
34	E_tor	3D	Torsion energy
35	GCUT_PEOE_3	2D	PEOE Charge GCUT (3/3)
36	GCUT_SLOGP_3	2D	LogP GCUT (3/3)
37	GCUT_SMR_3	2D	Molar Refractivity GCUT (3/3)
38	h_ema	2D	Sum of EHT acceptor strengths
39	h_emd	2D	Sum of EHT donor strengths
40	h_emd_C	2D	Sum of EHT carbon donor strengthsc'c'c'c
41	h_log_pbo	2D	Sum of log (1 + p-bond orders)
42	h_mr	2D	Molar Refractivity
43	Kier1	2D	First kappa shape index
44	Kier2	2D	Second kappa shape index
45	Kier3	2D	Third kappa shape index
46	KierA1	2D	First alpha modified shape index
47	KierA2	2D	Second alpha modified shape index
48	KierA3	2D	Third alpha modified shape index
49	KierFlex	2D	Molecular flexibility
50	lip_acc	2D	Lipinski Acceptor Count
51	lip_don	2D	Lipinski Donor Count
52	logS	2D	Log Solubility in Water
53	mr	2D	Molar refractivity
54	opr_brigid	2D	Oprea Rigid Bond Count
55	opr_nrot	2D	Oprea Rotatable Bond Count
56	PEOE_PC+	2D	Total positive partial charge
57	PEOE_PC-	2D	Total negative partial charge

58	PEOE_RPC+	2D	Relative positive partial charge
59	PEOE_RPC-	2D	Relative negative partial charge
60	PEOE_VSA+1	2D	Total positive 1 vdw surface area
61	PEOE_VSA+2	2D	Total positive 2 vdw surface area
62	PEOE_VSA+3	2D	Total positive 3 vdw surface area
63	PEOE_VSA+4	2D	Total positive 4 vdw surface area
64	PEOE_VSA-1	2D	Total negative 1 vdw surface area
65	PEOE_VSA-5	2D	Total negative 5 vdw surface area
66	PEOE_VSA_HYD	2D	Total hydrophobic vdw surface area
67	PEOE_VSA_NEG	2D	Total negative vdw surface area
68	PEOE_VSA_PNEG	2D	Total polar negative vdw surface area
69	PEOE_VSA_POL	2D	Total polar vdw surface area
70	PEOE_VSA_POS	2D	Total positive vdw surface area
71	PEOE_VSA_PPOS	2D	Total polar positive vdw surface area
72	pmi	3D	Principal moment of inertia
73	pmi1	3D	Principal moment of inertia (1)
74	pro_asa_hph	Protein	Hydrophilic Surface Area
75	pro_asa_vdw	Protein	Accessible Surface Area (Water Probe)
76	pro_coeff_diff	Protein	Diffusion Coefficient
77	pro_coeff_fric	Protein	Frictional Coefficient
78	pro_henry	Protein	Henry's Function f(ka)
79	pro_mass	Protein	Protein Mass in kDa
80	pro_r_solv	Protein	Hydrodynamic Radius
81	pro_sed_const	Protein	Sedimentation Constant
82	pro_volume	Protein	Protein Volume
83	Q_VSA_HYD	2D	Total hydrophobic vdw surface area
84	Q_VSA_POS	2D	Total positive vdw surface area
85	rings	2D	Number of rings
86	SlogP	2D	Log Octanol/Water Partition Coefficient
87	SlogP_VSA0	2D	Bin 0 SlogP (-10, -0.40]
88	SlogP_VSA1	2D	Bin 1 SlogP (-0.40, -0.20]
89	SlogP_VSA2	2D	Bin 2 SlogP (-0.20, 0.00]
90	SlogP_VSA3	2D	Bin 3 SlogP (0.00, 0.10]
91	SlogP_VSA5	2D	Bin 4 SlogP (0.10, 0.15]
92	SMR	2D	Molar Refractivity
93	SMR_VSA0	2D	Bin 0 SMR (0.000,0.110]
94	SMR_VSA2	2D	Bin 2 SMR (0.260,0.350]
95	SMR_VSA3	2D	Bin 3 SMR (0.350,0.390]
96	SMR_VSA4	2D	Bin 4 SMR (0.390,0.440]
97	SMR_VSA5	2D	Bin 5 SMR (0.440,0.485]
98	SMR_VSA6	2D	Bin 6 SMR (0.485,0.560]
99	std_dim2	3D	Standard dimension 2
100	std_dim3	3D	Standard dimension 3
101	TPSA	2D	Topological Polar Surface Area (A**2)

102	VAdjEq	2D	Vertex adjacency information (equal)
103	VAdjMa	2D	Vertex adjacency information (mag)
104	VDistMa	2D	Vertex distance magnitude index
105	vdw_area	2D	Van der Waals volume (A**3)
106	vdw_vol	2D	Vertex distance magnitude index
107	vol	3D	Van der Waals volume
108	VSA	3D	Van der Waals surface area
109	vsa_acc	2D	VDW acceptor surface area (A**2)
110	vsa_don	2D	VDW donor surface area (A**2)
111	vsa_hyd	2D	VDW hydrophobe surface area (A**2)
112	vsa_other	2D	VDW other surface area (A**2)
113	vsa_pol	2D	VDW polar surface area (A**2)
114	vsurf_CP	3D	Critical packing parameter
115	vsurf_CW1	3D	Capacity factor at -0.2
116	vsurf_CW5	3D	Capacity factor at -3.0
117	vsurf_CW6	3D	Capacity factor at -4.0
118	vsurf_CW7	3D	Capacity factor at -5.0
119	vsurf_CW8	3D	Capacity factor at -6.0
120	vsurf_D8	3D	Hydrophobic volume at -1.6
121	vsurf_EWmin3	3D	3rd lowest hydrophilic energy
122	vsurf_HB5	3D	H-bond donor capacity at -3.0
123	vsurf_HB6	3D	H-bond donor capacity at -4.0
124	vsurf_HB7	3D	H-bond donor capacity at -5.0
125	vsurf_HB8	3D	H-bond donor capacity at -6.0
126	vsurf_HL1	3D	First hydrophilic-lipophilic balance
127	vsurf_HL2	3D	Second hydrophilic-lipophilic balance
128	vsurf_S	3D	Interaction field area
129	vsurf_V	3D	Interaction field volume
130	vsurf_W1	3D	Hydrophilic volume at -0.2
131	vsurf_W2	3D	Hydrophilic volume at -0.5
132	vsurf_W3	3D	Hydrophilic volume at -1.0
133	vsurf_W4	3D	Hydrophilic volume at -2.0
134	vsurf_W5	3D	Hydrophilic volume at -3.0
135	vsurf_W6	3D	Hydrophilic volume at -4.0
136	vsurf_W7	3D	Hydrophilic volume at -5.0
137	vsurf_W8	3D	Hydrophilic volume at -6.0
138	vsurf_Wp1	3D	Polar volume at -0.2
139	vsurf_Wp2	3D	Polar volume at -0.5
140	vsurf_Wp3	3D	Polar volume at -1.0
141	vsurf_Wp4	3D	Polar volume at -2.0
142	vsurf_Wp5	3D	Polar volume at -3.0
143	vsurf_Wp6	3D	Polar volume at -4.0
144	vsurf_Wp7	3D	Polar volume at -5.0
145	vsurf_Wp8	3D	Polar volume at -6.0

146	Weight	2D	Molecular weight (CRC)
147	weinerPol	2D	Weiner polarity number
148	zagreb	2D	Zagreb index

Table S3. 20 descriptors were obtained from D2 dataset through feature screening method of WEKA software.

Number	Feature	Class	Feature Description
1	b_1rotR	2D	Fraction of rotatable single bonds
2	b_rotR	2D	Fraction of rotatable bonds
3	E_ang	3D	Angle Bend Energy
4	E_oop	3D	Out-of-plane Energy
5	E_vdw	3D	Van der Waals energy
6	GCUT_SLOGP_2	2D	LogP GCUT (2/3)
7	h_logS	2D	Log solubility in water
8	opr_brigid	2D	Oprea Rigid Bond Count
9	PEOE_RPC+	2D	Relative positive partial charge
10	PEOE_RPC-	2D	Relative negative partial charge
11	PEOE_VSA+2	2D	Total positive 2 vdw surface area
12	PEOE_VSA-1	2D	Total negative 1 vdw surface area
13	PEOE_VSA-3	2D	Total negative 3 vdw surface area
14	PEOE_VSA_FPPOS	2D	Fractional polar positive vdw surface area
15	petitjean	2D	(diameter - radius) / diameter
16	vsurf_CW5	3D	Capacity factor at -3.0
17	vsurf_W5	3D	Hydrophilic volume at -3.0
18	vsurf_W7	3D	Hydrophilic volume at -5.0
19	vsurf_Wp2	3D	Polar volume at -0.5
20	vsurf_Wp8	3D	Polar volume at -6.0

Table S4. The D3 dataset has 37 suggested descriptors in the MOE.

Number	Feature	Class	Feature Description
1	b_max1len	2D	Maximum single-bond chain length
2	density	2D	Mass density (AMU/A**3)
3	E_ang	3D	Angle Bend Energy
4	E_oop	3D	Out-of-plane Energy
5	E_tor	3D	Torsion energy
6	GCUT_PEOE_0	2D	PEOE Charge GCUT (0/3)
7	PEOE_PC-	2D	Total negative partial charge
8	PEOE_VSA+1	2D	Total positive 1 vdw surface area
9	PEOE_VSA+2	2D	Total positive 2 vdw surface area
10	PEOE_VSA+3	2D	Total positive 3 vdw surface area
11	PEOE_VSA+4	2D	Total positive 4 vdw surface area
12	PEOE_VSA+5	2D	Total positive 5 vdw surface area
13	PEOE_VSA+6	2D	Total positive 6 vdw surface area
14	PEOE_VSA-1	2D	Total negative 1 vdw surface area

15	PEOE_VSA-2	2D	Total negative 2 vdw surface area
16	PEOE_VSA-4	2D	Total negative 4 vdw surface area
17	PEOE_VSA_FHYD	2D	Fractional hydrophobic vdw surface area
18	PEOE_VSA_FNEG	2D	Fractional negative vdw surface area
19	PEOE_VSA_FPNEG	2D	Fractional polar negative vdw surface area
20	PEOE_VSA_FPOL	2D	Fractional polar vdw surface area
21	PEOE_VSA_FPOS	2D	Fractional positive vdw surface area
22	PEOE_VSA_FPPOS	2D	Fractional polar positive vdw surface area
23	PEOE_VSA_NEG	2D	Total negative vdw surface area
24	rsynth	2D	Synthetic Feasibility
25	vsurf_CW3	3D	Capacity factor at -1.0
26	vsurf_CW4	3D	Capacity factor at -2.0
27	vsurf_EWmin1	3D	Lowest hydrophilic energy
28	vsurf_EWmin2	3D	2nd lowest hydrophilic energy
29	vsurf_EWmin3	3D	3rd lowest hydrophilic energy
30	vsurf_HB1	3D	H-bond donor capacity at -0.2
31	vsurf_HB2	3D	H-bond donor capacity at -0.5
32	vsurf_HB3	3D	H-bond donor capacity at -1.0
33	vsurf_R	3D	Surface rugosity
34	vsurf_Wp3	3D	Polar volume at -1.0
35	vsurf_Wp4	3D	Polar volume at -2.0
36	vsurf_Wp5	3D	Polar volume at -3.0
37	vsurf_Wp6	3D	Polar volume at -4.0

Table S5. 13 features were obtained from D3 dataset using WEKA feature screening method.

Number	Feature	Class	Feature Description
1	density	2D	Mass density
2	E_oop	3D	Out-of-plane Energy
3	E_str	3D	Bond stretch energy
4	E_vdw	3D	Van der Waals energy
5	GCUT_PEOE_0	2D	PEOE Charge GCUT (0/3)
6	GCUT_PEOE_2	2D	PEOE Charge GCUT (2/3)
7	PEOE_VSA+2	2D	Total positive 2 vdw surface area
8	PEOE_VSA+4	2D	Total positive 4 vdw surface area
9	PEOE_VSA-1	2D	Total negative 1 vdw surface area
10	vsurf_HB2	3D	H-bond donor capacity at -0.5
11	vsurf_Wp4	3D	Polar volume at -2.0
12	vsurf_Wp5	3D	Polar volume at -3.0
13	vsurf_Wp6	3D	Polar volume at -4.0

Table S6. The D4 dataset has 9 suggested descriptors in the MOE.

Number	Feature	Class	Feature Description
1	b_max1len	2D	Maximum single-bond chain length
2	density	2D	Mass density (AMU/A**3)
3	PEOE_VSA+1	2D	Total positive 1 vdw surface area
4	PEOE_VSA+4	2D	Total positive 4 vdw surface area
5	PEOE_VSA-1	2D	Total negative 1 vdw surface area
6	PEOE_VSA_FHYD	2D	Fractional hydrophobic vdw surface area
7	PEOE_VSA_FPNEG	2D	Fractional polar negative vdw surface area
8	PEOE_VSA_FPOL	2D	Fractional polar vdw surface area
9	vsurf_Wp3	3D	Polar volume at -1.0

Table S7. 14 features were obtained from D4 dataset using WEKA feature screening method.

Number	Feature	Class	Feature Description
1	density	2D	Mass density (AMU/A**3)
2	E	3D	Potential Energy
3	E_str	3D	Bond stretch energy
4	GCUT_PEOE_0	2D	PEOE Charge GCUT (0/3)
5	h_pstates	2D	Entropic state count (pH=7)
6	PEOE_VSA+2	2D	Total positive 2 vdw surface area
7	PEOE_VSA+4	2D	Total positive 4 vdw surface area
8	PEOE_VSA-1	2D	Total negative 1 vdw surface area
9	PEOE_VSA-6	2D	Total negative 6 vdw surface area
10	PEOE_VSA_FHYD	2D	Fractional hydrophobic vdw surface area
11	PEOE_VSA_FPNEG	2D	Fractional polar negative vdw surface area
12	vsurf_HB4	3D	H-bond donor capacity at -2.0
13	vsurf_Wp3	3D	Polar volume at -1.0
14	vsurf_Wp4	3D	Polar volume at -2.0

Table S8. The D5 dataset has 19 suggested descriptors in the MOE.

Number	Feature	Class	Feature Description
1	b_1rotR	2D	Fraction of rotatable single bonds
2	b_max1len	2D	Maximum single-bond chain length
3	b_rotR	2D	Fraction of rotatable bonds
4	E_tor	3D	Torsion energy
5	PEOE_PC+	2D	Total positive partial charge
6	PEOE_PC-	2D	Total negative partial charge
7	PEOE_VSA+1	2D	Total positive 1 vdw surface area
8	PEOE_VSA+3	2D	Total positive 3 vdw surface area
9	PEOE_VSA+4	2D	Total positive 4 vdw surface area
10	PEOE_VSA-1	2D	Total negative 1 vdw surface area
11	PEOE_VSA_FHYD	2D	Fractional hydrophobic vdw surface area
12	PEOE_VSA_FPOL	2D	Fractional polar vdw surface area

13	PEOE_VSA_FPPOS	2D	Fractional polar positive vdw surface area
14	PEOE_VSA_HYD	2D	Total hydrophobic vdw surface area
15	PEOE_VSA_NEG	2D	Total negative vdw surface area
16	vsurf_Wp2	3D	Polar volume at -0.5
17	vsurf_Wp3	3D	Polar volume at -1.0
18	vsurf_Wp4	3D	Polar volume at -2.0
19	vsurf_Wp5	3D	Polar volume at -3.0

Table S9. The remaining 17 features of D5 dataset were selected by WEKA feature screening method.

Number	Feature	Class	Feature Description
1	b_max1len	2D	Maximum single-bond chain length
2	b_rotR	2D	Fraction of rotatable bonds
3	E	3D	Potential Energy
4	GCUT_PEOE_0	2D	PEOE Charge GCUT (0/3)
5	opr_brigid	2D	Oprea Rigid Bond Count
6	PEOE_VSA+1	2D	Total positive 1 vdw surface area
7	PEOE_VSA+4	2D	Total positive 4 vdw surface area
8	PEOE_VSA-1	2D	Total negative 1 vdw surface area
9	PEOE_VSA-3	2D	Total negative 3 vdw surface area
10	PEOE_VSA-4	2D	Total negative 4 vdw surface area
11	PEOE_VSA_FNEG	2D	Fractional negative vdw surface area
12	PEOE_VSA_FPNEG	2D	Fractional polar negative vdw surface area
13	PEOE_VSA_FPPOS	2D	Fractional polar positive vdw surface area
14	SMR_VSA4	2D	Bin 4 SMR (0.390,0.440]
15	vsurf_CW4	3D	Capacity factor at -2.0
16	vsurf_HB7	3D	H-bond donor capacity at -5.0
17	vsurf_Wp4	3D	Polar volume at -2.0

Table S10. The natural hemolytic peptide dataset HD1 was screened by WEKA method with the remaining 7 features.

Number	Feature	Class	Feature Description
1	chi1v_C	2D	Carbon valence connectivity index (order 1)
2	logS	2D	Log Solubility in Water
3	PEOE_VSA-0	2D	Total negative 0 vdw surface area
4	SlogP_VSA4	2D	Bin 4 SlogP (0.10, 0.15]
5	SlogP_VSA9	2D	Bin 9 SlogP (0.40,10]
6	vsurf_CW4	3D	Capacity factor at -2.0
7	vsurf_CW5	3D	Capacity factor at -3.0

Table S11. 18 features were obtained from the chemically modified peptide dataset HD2 using WEKA feature screening method.

Number	Feature	Class	Feature Description
1	BCUT_PEOE_0	2D	PEOE Charge BCUT (0/3)
2	BCUT_SLOGP_3	2D	LogP BCUT (3/3)
3	b_rotN	2D	Number of rotatable bonds
4	GCUT_PEOE_2	2D	PEOE Charge GCUT (2/3)
5	logS	2D	Log Solubility in Water
6	PEOE_VSA+4	2D	Total positive 4 vdw surface area
7	petitjean	2D	(diameter - radius) / diameter
8	pro_helicity	Protein	Protein Helix Ratio
9	pro_hyd_moment	Protein	Hydrophobicity Moment
10	pro_patch_hyd	Protein	Area of hydrophobic protein patch(es)
11	pro_patch_hyd_1	Protein	Area of largest hydrophobic protein patch(es)
12	pro_patch_hyd_2	Protein	Area of 2 largest hydrophobic protein patch(es)
13	pro_patch_hyd_4	Protein	Area of 4 largest hydrophobic protein patch(es)
14	SlogP_VSA4	2D	Bin 4 SlogP (0.10, 0.15]
15	SMR_VSA7	2D	Bin 7 SlogP (0.25, 0.30]
16	vsurf_CW1	3D	Capacity factor at -0.2
17	vsurf_HB1	3D	H-bond donor capacity at -0.2
18	vsurf_HB5	3D	H-bond donor capacity at -3.0

Table S12. In the mixed hemolytic peptide dataset HD3, WEKA feature screening method was used to obtain 18 features.

Number	Feature	Class	Feature Description
1	BCUT_PEOE_0	2D	PEOE Charge BCUT (0/3)
2	BCUT_SLOGP_3	2D	LogP BCUT (3/3)
3	BCUT_SMR_3	2D	Molar Refractivity BCUT (3/3)
4	E_ele	3D	Electrostatic energy
5	h_logS	2D	Log solubility in water
6	Kier3	2D	Third kappa shape index
7	logP(o/w)	2D	Log octanol/water partition coefficient
8	logS	2D	Log Solubility in Water
9	PEOE_VSA+4	2D	Total positive 4 vdw surface area
10	PEOE_VSA-1	2D	Total negative 1 vdw surface area
11	PEOE_VSA_NEG	2D	Total negative vdw surface area
12	pro_helicity	Protein	Protein Helix Ratio
13	pro_patch_hyd	Protein	Area of hydrophobic protein patch(es)
14	pro_patch_hyd_1	Protein	Area of largest hydrophobic protein patch(es)
15	pro_patch_hyd_2	Protein	Area of 2 largest hydrophobic protein patch(es)
16	pro_patch_hyd_4	Protein	Area of 4 largest hydrophobic protein patch(es)
17	SlogP_VSA4	2D	Bin 4 SlogP (0.10, 0.15]
18	SlogP_VSA9	2D	Bin 9 SlogP (0.40,10]

19	SMR_VSA7	2D	Bin 7 SMR (0.560,10]
20	vsa_hyd	2D	VDW hydrophobe surface area (A**2)
21	vsurf_CW5	3D	Capacity factor at -3.0
22	vsurf_HB1	3D	H-bond donor capacity at -0.2
23	vsurf_HB5	3D	H-bond donor capacity at -3.0

Table S13. 22 descriptors were obtained from the natural toxic peptide dataset TD1 by WEKA feature screening method.

Number	Feature	Class	Feature Description
1	a_ICM	2D	Atom information content (mean)
2	BCUT_PEOE_1	2D	PEOE Charge BCUT (1/3)
3	BCUT_PEOE_3	2D	PEOE Charge BCUT (3/3)
4	BCUT_SLOGP_0	2D	LogP BCUT (0/3)
5	bpol	2D	Difference of bonded atom polarizabilities
6	E	3D	Potential Energy
7	GCUT_PEOE_0	2D	PEOE Charge GCUT (0/3)
8	GCUT_PEOE_1	2D	PEOE Charge GCUT (1/3)
9	h_pavgQ	2D	Average total charge (pH=7)
10	Kier3	2D	Third kappa shape index
11	PEOE_VSA+0	2D	Total positive 0 vdw surface area
12	PEOE_VSA-1	2D	Total negative 1 vdw surface area
13	PEOE_VSA-3	2D	Total negative 3 vdw surface area
14	PEOE_VSA-5	2D	Total negative 5 vdw surface area
15	PEOE_VSA_PPOS	2D	Total polar positive vdw surface area
16	pro_helicity	Protein	Protein Helix Ratio
17	pro_mobility	Protein	Protein Mobility
18	pro_pI_seq	Protein	Sequence-based pI Prediction
19	pro_volume	Protein	Protein Volume
20	SlogP_VSA1	2D	Bin 1 SlogP (-0.40, -0.20]
21	SlogP_VSA4	2D	Bin 4 SlogP (0.10, 0.15]
22	SMR_VSA6	2D	Bin 6 SlogP (0.20, 0.25]

Table S14. In the toxic peptide dataset TD2, 15 descriptors were obtained by WEKA feature screening.

Number	Feature	Class	Feature Description
1	a_don	2D	Number of H-bond donor atoms
2	a_nN	2D	Number of nitrogen atoms
3	b_1rotR	2D	Fraction of rotatable single bonds
4	h_logS	2D	Log solubility in water
5	PEOE_VSA+2	2D	Total positive 2 vdw surface area
6	pro_app_char	Protein	Protein Charge at Debye Length
7	pro_asa_hyd	Protein	Hydrophobic Surface Area
8	pro_patch_hyd	Protein	Area of hydrophobic protein patch(es)
9	vsa_pol	2D	VDW polar surface area (A**2)

10	vsurf_A	3D	Amphiphilic moment
11	vsurf_CW1	3D	Capacity factor at -0.2
12	vsurf_EDmin1	3D	Lowest hydrophobic energy
13	vsurf_EDmin3	3D	3rd lowest hydrophobic energy
14	vsurf_ID2	3D	Hydrophobic integy moment at -0.4
15	vsurf_ID7	3D	Hydrophobic integy moment at -1.4

Table S15. In the mixed toxic peptide dataset TD3, there were 27 descriptors remaining using WEKA feature screening method.

Number	Feature	Class	Feature Description
1	a_donacc	2D	Number of H-bond donor + acceptor atoms
2	a_ICM	2D	Atom information content (mean)
3	BCUT_PEOE_1	2D	PEOE Charge BCUT (1/3)
4	BCUT_PEOE_3	2D	PEOE Charge BCUT (3/3)
5	BCUT_SLOGP_0	2D	LogP BCUT (0/3)
6	bpol	2D	Difference of bonded atom polarizabilities
7	b_count	2D	Number of bonds
8	E	3D	Potential Energy
9	GCUT_PEOE_1	2D	PEOE Charge GCUT (1/3)
10	GCUT_SLOGP_0	2D	LogP GCUT (0/3)
11	h_pavgQ	2D	Average total charge (pH=7)
12	Kier3	2D	Third kappa shape index
13	PEOE_VSA+0	2D	Total positive 0 vdw surface area
14	PEOE_VSA-1	2D	Total negative 1 vdw surface area
15	PEOE_VSA-3	2D	Total negative 3 vdw surface area
16	PEOE_VSA-5	2D	Total negative 5 vdw surface area
17	PEOE_VSA_PPOS	2D	Total polar positive vdw surface area
18	pro_helicity	Protein	Protein Helix Ratio
19	pro_mobility	Protein	Protein Mobility
20	pro_patch_hyd_1	Protein	Area of largest hydrophobic protein patch(es) near CDRs
21	pro_pI_seq	Protein	Sequence-based pI Prediction
22	pro_volume	Protein	Protein Volume
23	pro_zeta	Protein	Zeta potential at Debye Length
24	SlogP_VSA1	2D	Bin 1 SlogP (-0.40, -0.20]
25	SlogP_VSA4	2D	Bin 4 SlogP (0.10, 0.15]
26	SMR_VSA4	2D	Bin 4 SMR (0.390,0.440]
27	vsurf_DD12	3D	vsurf_EDmin1, vsurf_EDmin2 distance

Table S16. Sequence information of 41 candidate ACPs.

ID	SATPdb	Name	Sequence
1	15988		GLLSGTSVRGST
2	16053	Brevinin-2LF2	SIMSTLKQFGISAIKGAAQNVLGVLSCKIAKTC
3	16563	RANATUERIN-2Lb	GILSSIKGVAKGVAKNVAAQLLDTLKCKITGC
4	17354	Brevinin-2	GLLDSLKGFAATAGKGVLQSLLSTASCKLAKTC
5	17502	Peptide 5	SPPSEQLGKSFNF
6	17633	Peptide 8	APPPGYAMESDSFS
7	17860	Pleurain-C1 (Fraction)	YPELQQDLIARL
8	17870	Thermophilin 9	LSCDEGMLAVGGLGAVGGPWGAAVGVLVGAALYCF
9	17899	Pleurain-M1	GLLDSVKEGLKKVAGQLLDTLKCKISGCTPA
10	18175	Caerulein precursor-related fragment Eb	GLGSFLKNAIKIAGKVGSTIGKVADAIGNKE
11	18394	Brevinin-2HSa	GLLDSLKNLAINAAKGAGQSVLNTLSCKLSKTC
12	18475	Caerulein precursor-related fragment Ea	GLGSILGKILNVAGKVGKTIGKVADAVGNKE
13	18980	EP-20	EGPVGLADPDGPASAPLGAP
14	18988	Brevinin-2-OR5	SFLDTLKNLAISAAKGAGQSVLSTLSCKLSETC
15	19056	Silkworm 001	YGQSTHAVIYAQGYTYSSDWR
16	19414	Secretolytin	QKIAEKFSGTRRG
17	19566	Brevinin-2DYd	GIFDVVKGVLKGVGKNVAGSLLEQLKCKLSGGC
18	20815	Nigrocin-1	GLLDSIKGMAISAGKGALQNLLKVASCKLDKTC
19	21972	Thurincin H	DWTCWSCLVCAACSVELLNLVTAATGASTAS
20	22121	Odorranain-C1	GVLGAVKDLLIGAGKSAAQSVLKTLSCKLSNDC
21	22355	RANATUERIN 2	GLFLDTLKGAAKDVAGKLEGLKCKITGCKLP
22	22826	Shuchin 2	NALSSPRNKCDRASSCFG
23	23457	Acidocin J1132	NPKVAHCASQIGRSTAWGAVSGA
24	23861	So-D1	TCESPSHKFKGPCATNRNCES
25	23899	Acidocin J1132 β	GNPKVAHCASQIGRSTAWGAVSGA
26	24054	Formaecin-1(Fraction)	GRPNPVNNKPTPHPR
27	24261	Brevinin-2-OA7	GVLGTVKDLLIGAGKSAAQSTLKTLSCKISNDC
28	24656	Astexin-1	GLSQGVEPDIGQTYFEESRINQD
29	24914	Pyrrhocoricin	VDKGSYLPRPTPPRPIYNRN
30	25276	Tricholongin BI(Fraction)	AGFAAQAAASLAPVAAQQ
31	25797	Histone H6-like protein	PKRKSATKGDEPA
32	25868	Shepherin I	GYGGHGGHGGHGGHGGHGGHGHGGGGHG
33	26074	Brevinin-2RTa	GLMSTLKDFGKTAAKEIAQSLLSTASCKLAKTC
34	26753	Brevinin-2EA	GILDTLKNLAISAAKGAAQGLVNKASCKLSGQC
35	27223	Brevinin-2-OA5	GLLDGILNANFNAAKSAGTSVLNALSCKLSKTC
36	27375	Ranatuerin-2G	GLLLDTLKGAAKDIAGIALEKLKCKITGCKP
37	27529	Planosporicin	ITSVSWCTPGCTSEGGGSGCSHCC
38	27843	Brevinin-2DYb	GLFDVVKGVLKGAGKNVAGSLLEQLKCKLSGGC

39	28633	Brevinin-2HS2	SLLGTVKDLLIGAGKSAAQSVLKGLSGKLSKDC
40	28961	Caerulein precursor-related fragment Ec	GLGSFFKNAIKIAGKVGSTIGKVADAIGNKE
41	28962	Palustrin-2a	GFLSTVKNLATNVAGTVLDTIRCKVTGGCRP
